# When Patients Recover From COVID-19: Data-Driven Insights From Wearable Technologies

**DOI:** 10.3389/fdata.2022.801998

**Published:** 2022-04-28

**Authors:** Muzhe Guo, Long Nguyen, Hongfei Du, Fang Jin

**Affiliations:** ^1^Department of Statistics, The George Washington University, Washington, DC, United States; ^2^Department of Computer Science and Data Science, School of Applied Computational Sciences, Meharry Medical College, Nashville, TN, United States

**Keywords:** COVID-19, wearable data, neural networks, uncertainty quantification, pattern extraction

## Abstract

Coronavirus disease 2019 (COVID-19) is known as a contagious disease and caused an overwhelming of hospital resources worldwide. Therefore, deciding on hospitalizing COVID-19 patients or quarantining them at home becomes a crucial solution to manage an extremely big number of patients in a short time. This paper proposes a model which combines Long-short Term Memory (LSTM) and Deep Neural Network (DNN) to early and accurately classify disease stages of the patients to address the problem at a low cost. In this model, the LSTM component will exploit temporal features while the DNN component extracts attributed features to enhance the model's classification performance. Our experimental results demonstrate that the proposed model achieves substantially better prediction accuracy than existing state-of-art methods. Moreover, we explore the importance of different vital indicators to help patients and doctors identify the critical factors at different COVID-19 stages. Finally, we create case studies demonstrating the differences between severe and mild patients and show the signs of recovery from COVID-19 disease by extracting shape patterns based on temporal features of patients. In summary, by identifying the disease stages, this research will help patients understand their current disease situation. Furthermore, it will also help doctors to provide patients with an immediate treatment plan remotely that addresses their specific disease stages, thus optimizing their usage of limited medical resources.

## 1. Introduction

Coronavirus disease 2019 (COVID-19), which is caused by severe acute respiratory syndrome coronavirus 2 (SARS-CoV-2), manifests as a wide range of symptoms, including fever, cough, fatigue, breathing difficulties, loss of smell and taste, and pneumonia^1^. It spreads rapidly from infected people to others through close contact or small exhaled droplets. The pandemic is now causing havoc in countries around the world, with more than 282 million cases and around 5.41 million deaths, as of late December 2021 reported by WHO (2021). This deluge of patients is overwhelming hospitals everywhere, especially in some developing countries where vaccines are not sufficient, and it is difficult to cope with the need to conduct extensive disease testing programs and treat huge numbers of patients in a very short period. It is therefore vital for medical staff to be able to identify patients COVID-19 disease stages before making the decision to hospitalize them. Severe patients need to be hospitalized quickly and receive a higher priority in dedicated treatment, while patients with milder symptoms might only need to self-quarantine at home. Fast and reliable techniques to detect and identify the disease stages are thus the focus of active research by scientists and medical technologists.

Vaira et al. found that anosmia and ageusia associated with fever (>37.5°C) are common onset symptoms that can be an early signal of a COVID-19 infection (discussed by Heerfordt and Heerfordt, 2020; Ortiz-Martínez et al., 2020; Vaira et al., 2020; Walker et al., 2020), therefore, investigated the use of Google Trends to study the loss of smell and smoking cessation and predicted COVID-19 incidence. Wang et al. (2020) built a deep convolutional neural network model to detect COVID-19 from chest X-ray images. Most of the existing work focused on early disease detection, but few works were proposed to identify the disease stages and develop useful insights for patients who must quarantine at home. We therefore propose to explore the problem of disease stage identification, because this will help doctors decide the most appropriate treatment plans for patients at each stage, allowing them to optimize their usage of scarce resources when the hospital is under pressure. Besides, since our work would help create a low-cost, efficient self-monitor solution that can be used by everyone, it is beneficial, especially for people who are quarantined at home.

Interestingly, there have been some huge improvements in wearable technologies over the last few years, with a number of wearable devices being widely introduced that enhance our everyday life. For example, smartwatches such as Fitbit^2^ are helping us to track our sleep patterns and daily activities, encouraging us to maintain a healthier lifestyle. Smart Shirt is another example of this trend that is beginning to play an important role in our information infrastructure, supporting healthcare systems for monitoring vital signs efficiently and cost-effectively with the universal interface of clothing (Park and Jayaraman, 2003). The possibilities are seemingly unlimited: chip-integrated sensors are being used to monitor a number of physical medicine applications (Bonato, 2005). Sensors have already been developed specifically for COVID-19 applications, including an automatic sanitizer tunnel that detects a human being using an ultrasonic sensor from a distance of 1.5 feet and disinfects him/her using a sanitizer spray (Pandya et al., 2020). Quer et al. (2020) used wearable sensors to differentiate COVID-19 positive vs. negative cases in symptomatic individuals, pointing out that wearable devices are easy to access for most people. The fast development of wearable technologies makes it possible to be utilized to identify COVID-19 disease stages. However, existing studies are all either (i) mainly limited to the detection of COVID-19, with no attempt to identify the stages of the disease; (ii) not designed to analyze variations in the associated factors per COVID-19 stage; or (iii) unable to provide a comprehensive view of the disease for layman readers. Therefore, we seized this opportunity to investigate data-driven approaches to COVID-19 through wearable technologies in an attempt to bridge this gap. This paper introduces a wide-ranging set of data-driven approaches to identify infected patients' stages using wearable technologies. Specifically, this work aims to accurately and early infer from wearable data obtained from sensing devices attached to COVID-19 patients whether the COVID-19 patients are in mild, moderate, severe, or recovery stages in an earlier stage. We achieved this by introducing a model that utilizes a Long-short Term Memory (LSTM) network and a Deep Neural Network (DNN) to aggregate and jointly exploit temporal stream data from wearable devices and attribute stream from characteristics of patients. It is worth mentioning that our comprehensive experimental evaluation shows the improved performance achieved by our model compared to existing machine learning (ML) classification methods, which can only use one of the data streams. By identifying these patients in earlier stages, medical professionals will be able to take swift action if the patient requires early hospitalization or if it is safe for them to continue to self-quarantine at home. In addition, we also compare the lifestyles between severe and mild patients, allowing us to investigate and evaluate factors that impact the recovery of the patients. Specifically, the work aims to address the following three research questions (**RQs**):

- **RQ1:** Can we build an accurate ML model to predict COVID-19 stages and identify whether a patient will progress to a more severe stage in an earlier stage?
- **RQ2:** Which set of factors are associated with the severity of a patients symptoms? What can we learn from these factors in association with COVID-19 stages?
- **RQ3:** What signs signify recovery or deterioration in COVID-19 patients?

Overall, three novel contributions are made in this research:

1. We develop a classification model with uncertainty quantification to identify the major COVID-19 disease stages. Our model is able to recognize patients' disease stages in a timely manner because we utilize data from the wearable device, which is more responsive to disease stages than the subject's senses.
2. Our work provides useful insights into the progression of COVID-19 disease and vital indicators at each stage. The research input is from a data source (a wearable device like a smartwatch) that everyone can access and use on their own. Our approach is data-driven and can mitigate human bias substantially.
3. We investigate factors associated with COVID-19 severity and recovery. We also create case studies (1) demonstrating the differences between severe and mild patients and (2) showing the signs of recovery from COVID-19 disease using a shape-based pattern extraction model.

The rest of this paper is organized as follows. Section 2 reviews the related work. Section 3 discusses our methodology, including an overview of the data preparation, stage identification model, feature importance, and pattern extraction model. Section 4 shows our evaluation and experimental results. Section 5 presents some limitation in our study. Finally, we offer conclusions in Section 6.

## 2. Related Work

Here, we survey recent related studies on battling the COVID-19 crisis. These studies fall into two broad scientific areas: machine learning (ML) and remote monitoring utilizing the Internet of Things (IoT).

**ML Research:** Researchers have attempted different methods to battle COVID-19. Assaf et al. developed a model that used white blood cell count, time from symptoms to admission, oxygen saturation, and blood lymphocyte count to predict if a patient is at high risk for COVID-19. Their prediction model can be useful for efficient triage and in-hospital allocation, better prioritization of medical resources, and improving overall management (Assaf et al., 2020). Ahamad et al. (2020) developed a model that applies ML algorithms to reveal potential COVID-19 patients by analyzing their age, gender, fever, and history of travel. By extracting 11 blood indices through a random forest algorithm, Wu et al. (2020) built an assistant discrimination tool that can identify suspected patients using their blood test results. Barstugan et al. (2020) and Elaziz et al. (2020) choose to use image-based diagnosis (CT images) building Support Vector Machine and K-Nearest Neighbors algorithms for predicting suspected COVID-19 infection.

**Remote monitoring research:** However, these studies' data sources, such as CT images or blood test results, would often need to be collected by trained professionals. With COVID-19 patients number rising, we see a shortage of medical resources worldwide and make clinic visits bear more risk as suspected patients gather for examination. Therefore, many people prefer to use the Internet of Things (IoT) to diagnose COVID-19 to avoid the risk of infection. Singh et al. demonstrated that IoT implementation could help infected patients with COVID-19 identify symptoms rapidly and greatly reduce healthcare costs (Singh et al., 2020). Islam et al. (2020) suggested that wearable devices could provide real-time remote monitoring and contact tracing features, which can be used to improve healthcare systems' current management schemes. For example, Maghdid et al. (2020) designed an artificial intelligence-enabled framework that analyzes signals from a smartphone's sensor signal. It helped to diagnose the severity of pneumonia to predict the COVID-19 infection.

Most prior works were focusing on the early prediction or detection of COVID-19 infection. As the epidemic escalates dramatically every day, we want to further conserve healthcare resources by identifying different stages of COVID-19 patients. For example, diagnosed early and moderate stage patients could adopt self-quarantine treatment in time, saving valuable resources that can then be utilized by patients with severe COVID-19 stage.

## 3. Method

### 3.1. Data Preparation

#### 3.1.1. Dataset Description

We used an open dataset provided by Welltory ^3^ The dataset comprises multivariate data records from 186 COVID-19 patients experiencing different stages. The data includes variables such as heart rate, sleeping patterns, daily activities, heart rate variability (HRV), blood pressure, patient demographics (age, gender, country, etc.), environmental information, and other patient facts (smoking, alcohol, other background diseases, etc.). We focus on the HRV information measured using wearable devices. HRV is also popular in many clinical and investigational research such as diabetes (Benichou et al., 2018), brain emotion, stress, anxiety (Goessl et al., 2017; Mather and Thayer, 2018), or cardiology related (Sessa et al., 2018). Table 1 provides detailed descriptions of HRV specific features, where rr_data (intervals in milliseconds between consecutive heartbeats) is a sequence data with a length of 100. In addition, we also selected ordered categorical variables with values from 1 to 6 recording the intensity of seven common COVID-19 symptoms that were in the HRV survey dataset: breath, confusion, cough, fatigue, fever, pain, and bluish. We believe these variables can better assist in the task of prediction, but we only focus on the other HRV variables for the subsequent analysis.

**Table 1:** List of features specific to heart rate variability (HRV).

**Feature name**	**Meaning**
bpm	Heart rate
mxdmn	Difference between highest and lowest cardio interval values
sdnn	Standard deviation of normal heartbeat intervals
rmssd	Root mean square of successive differences for consecutive intervals
pnn50	Percent of RR-intervals that fall outside a 50 ms range of the average
mode	Most common cardio interval length
amo	Mode amplitude
lf	Power of low frequency waves
hf	Power of high frequency waves
vlf	Power of very low frequency waves
lfhf	Ratio of low to high frequency waves
total_power	Total power of HF, LF, and VLF waves generated by the heart
rr_data (time-based)	Intervals in milliseconds between consecutive heart beats

**Figure 1:**
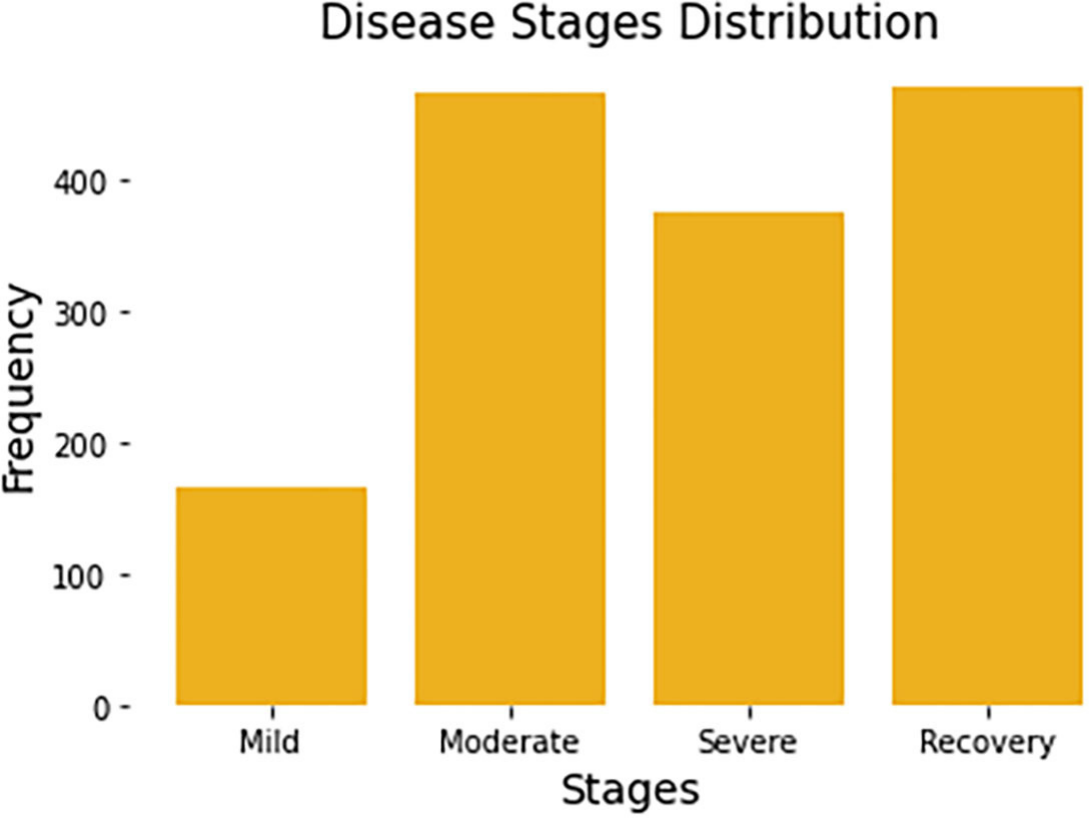
Distribution of four disease stages. Each patient may span multiple disease stages due to the progression of the disease.

Since each patient may be recorded multiple times, the stage of disease may be different from one recording period to the next. For example, some patients who were mild patients at the beginning of the record may become severe patients a week later. So, in the task of predicting the stage of disease, we remove the user code and predict the disease status for each record. All patients have a total of 1,480 complete records. Each record will be associated with a label by a survey from Welltory, identifying the corresponding patient's current stage. Figure 1 summarizes the number of stages per disease stage category.

#### 3.1.2. Feature Expansion

To make the most of the information in the data, we enrich our feature set based on temporal and statistical properties. First, for the variable time series, intervals in milliseconds between consecutive heartbeats (represented by *rr*), we computed a variety of statistics for this sequence, such as its variance (*rr*_*var*), skewness (*rr*_*skew*), kurtosis (*rr*_*kurt*), maximum (*rr*_*max*), minimum (*rr*_*min*), median (*rr*_*median*), mean (*rr*_*mean*), interquartile range (*rr*_*iqr*), etc. These features are popular and widely used in many research such as heart rate analysis (Bolanos et al., 2006) or brain waves recognition (Campisi and La Rocca, 2014). Besides, we divide each day into four periods and further create four one-hot variables: *morning*, *day*, *evening*, and *night*. That is, if a row of data for a patient is recorded in the morning, then the variable *morning* for this record is 1, while the other three variables are all 0. Another variable we created is called *day*_*after*_*test* (*days a*.*t*.), and its value depends on the number of days each patient has been infected with COVID-19.

In addition, we obtain two new temporal sequence data using the transformation of *rr*_*data*. Suppose the original heartbeat interval is *RR* = {*x*_1_, *x*_2_, ..., *x*_*T*_}, we transform this time series by computing lag difference (*DI*) and the absolute deviation from the mean (*DM*), in order to remove temporal dependency and to eliminate the trend and seasonality of the time series. Mathematically, the two newly constructed time-series are as follows:


(1) $$\begin{array}{l} DI=\left\{x_{2}-x_{1},x_{3}-x_{2},...,x_{T}-x_{T-1}\right\},\\ DM=\left\{\left|x_{1}-\bar{x}|,|x_{2}-\bar{x}|,...,|x_{T}-\bar{x}\right|\right\}, \end{array}$$

where *T* = 100 and $\bar{x}$ is the mean of the original *rr* sequence. To make these three sequences (*RR*, *DI*, and *DM*) have the same length 100, we add the average of the last three numbers of the DI sequence at the end of the DI sequence. All the features we expanded are listed in Table 2. Thus, we end up with a total of 32 attribute features and 3 temporal features for the task of predicting disease stages.

**Table 2:** List of self-generated features (time-based and statistical features).

**Domain**	**Feature name**	**Source**
Time-based	*DI*	Lag difference of *rr* sequence
	*DM*	Absolute deviation from the mean of *rr* sequence
Statistical	*rr*_*var*	Variance of *rr* sequence
	*rr*_*skew*	Skewness of *rr* sequence
	*rr*_*kurt*	Kurtosis of *rr* sequence
	*rr*_*max*	Maximum of *rr* sequence
	*rr*_*min*	Minimum of *rr* sequence
	*rr*_*median*	Median of *rr* sequence
	*rr*_*mean*	Mean of *rr* sequence
	*rr*_*iqr*	Interquartile range of *rr* sequence
	*morning*	One-hot variables *I*_*morning*_
	*day*	One-hot variables *I*_*day*_
	*evening*	One-hot variables *I*_*evening*_
	*night*	One-hot variables *I*_*night*_
	*daysa*.*t*.	Number of days of COVID-19 infection

#### 3.1.3. Data Pre-processing

There are some missing values in the dataset. It is either due to the network issues when the data is collected or the users choose not to answer some survey questions for any reason. To fill out the missing values, we used MissForest (Stekhoven and Bühlmann, 2012), a non-parametric iterative imputation technique based on the Random Forest algorithm which is proved capable of handling missing values of different data types. Additionally, we normalized the data to avoid scales influencing between features. Let *min*{*X*_*i*, 1:*N*_} and *max*{*X*_*i*, 1:*N*_} are the minimum and maximum values of the attribute feature *X*_*i*_ for all N samples. The min-max normalization values of feature *X*_*i*_ is computed as follows:


(2) $$\begin{array}{l} X_{i,j}^{\prime }=\frac{X_{i,j}-min\left\{X_{i,1:N}\right\}}{max\left\{X_{i,1:N}\right\}-min\left\{X_{i,1:N}\right\}}\;,\;j=1,2,...,N \end{array}$$

Where *N* = 1, 480 is the sample size.

Similarly, for the temporal sequence features, we use min-max normalization to normalize the data for all samples at each time point. Let *min*{*X*_*k, t*, 1:*N*_} and *max*{*X*_*k, t*, 1:*N*_} are the minimum and maximum values of the temporal feature *X*_*k*_ for all N samples at time *t*. The min-max normalization values of feature *X*_*k*_ is computed as:


(3) $$\begin{array}{r} X_{k,t,j}^{\prime }=\frac{X_{k,t,j}-min\left\{X_{k,t,1:N}\right\}}{max\left\{X_{k,t,1:N}\right\}-min\left\{X_{k,t,1:N}\right\}}\;,\;j=1,2,...,N,\\ \;t=1,2,...,T \end{array}$$

Where *N* = 1,480 is the sample size and *T* = 100 is the length of the temporal sequence.

### 3.2. Model for Disease Stage Identification

#### 3.2.1. Theoretical Model

We formulate the problem of identifying disease stages as a multi-class classification problem. From a feature matrix *X* of a patient, we need to build a classifier *f* that classifies whether the patient is in *Mild, Moderate, Severe*, or *Recovery* stage.

In this task, our classification model utilizes two data streams described in Section 3.1: *temporal stream* and *attribute stream*. A temporal stream has temporal characteristics or sequential order. The temporal streams can be real-time, so if our model is embedded in wearable devices in the future, it will be very helpful for early-stage detection. The attribute stream has no temporal characteristics such as demographic information, patient's background disease, etc. Formally, assume that the dataset D of size *N* is defined as D = {(*X*_*i*_, *Y*_*i*_), *i* = 1, ..., *N*}, where *Y*_*i*_ is the class label and $X_{i}=\left(X_{i}^{t},X_{i}^{a}\right)$, represents the i-th sample of the combination of the temporal stream (denoted as *X*^*t*^) and attribute stream (denoted as *X*^*a*^). The developed classification model *f* parameterized by θ will classify disease stages based on input streams as the following equation:


(4) $$\begin{array}{l} stages≃f\left(\theta ,H_{2}\left(\Phi \left(H_{1}\left(X^{t}\right),X^{a}\right)\right)\right), \end{array}$$

where *H*_1_ and *H*_2_ are latent feature extractors, which are two types of neural networks in our model, Φ is an aggregation function that fuses the latent features from $H_{1}\left(X^{t}\right)$ with attribute stream data *X*^*a*^.

#### 3.2.2. Network Design and Data Fusion Strategy

As mentioned earlier, the two input streams of the model are the temporal stream and the attribute stream. The LSTM network is suitable for temporal stream since it is a type of recurrent neural network (RNN) and addresses the problems of vanishing and exploding gradient in general RNNs. Hochreiter (1998). Therefore, in Equation (4), we choose *H*_1_ as an LSTM based network to learn latent features from the temporal stream *X*^*t*^. For the attribute stream *X*^*a*^, after combining them with the outputs of the LSTM based network, we use *H*_2_, a network of multiple fully-connected layers (DNN), to extract their latent features for the final disease stage classification. The DNN is chosen to force the network to explore all the possible relationships of both attribute streams and temporal streams. This is also an approach to combining DNN with LSTM to obtain a novel end-to-end neural network.

**Figure 2:**
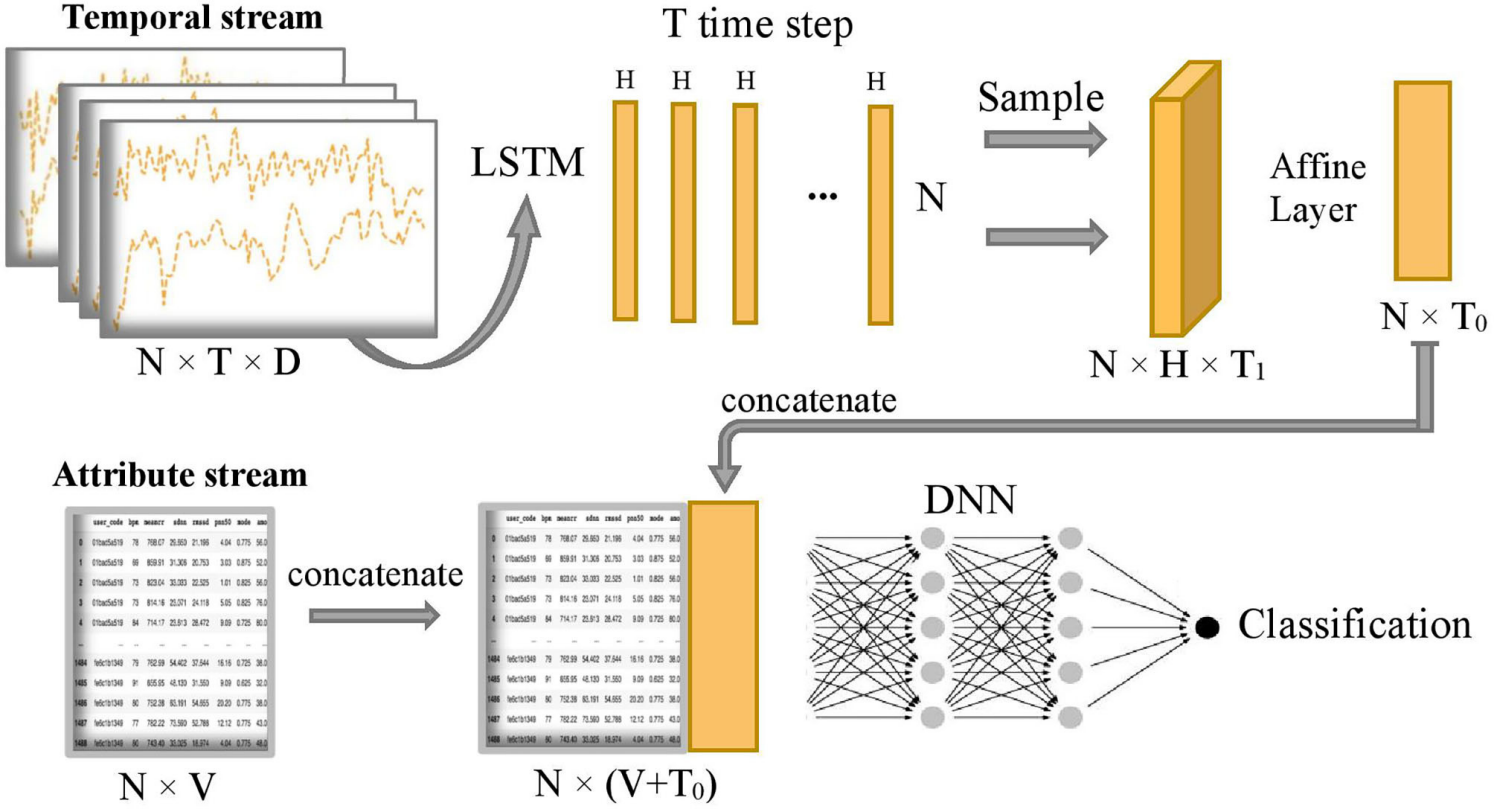
Overview of COVID-19 stage classification model, where N=1,480 represents the sample size, T=100 represents the common length of temporal sequence, D=3 represents the number of temporal sequences, H=1 represents the size of hidden state output by LSTM, T_1_=20 represents sampling size of the T hidden states, T_0_=5 represents the final projected size of temporal features in the time dimension, and V=32 represents the number of attribute features.

Figure 2 shows the overall model which composes of two subnetworks, LSTM and DNN. The two subnetworks are merged to predict the final disease stages. Suppose each patient has D input sequences with a common time length T. An LSTM passes forward over the entire temporal data sequences. We use the hidden size *H* = 1 in the LSTM, so later we can use an affine layer to map the hidden outputs to one-dimensional data of the same dimensional size as the attribute data. The LSTM unit is composed of a cell state *c*_*t*_, a so-called memory cell, a hidden state *h*_*t*_, an input gate *i*, a forget gate *f*, an output gate *o*, and an input modulation gate *g*. They are called gates because they control the flow through the LSTM. The four gates will be computed at each time step for cell and hidden state updates. The following is the outline formula of LSTM:


(5) $$\begin{array}{l} \left(\begin{array}{c} i\\ f\\ o\\ g \end{array}\right)=\left(\begin{array}{c} \sigma \\ \sigma \\ \sigma \\ \tanh \end{array}\right)W\left(\begin{array}{c} h_{t-1}\\ x_{t} \end{array}\right)\\ \;c_{t}=f⊙c_{t-1}+i⊙g\\ \;h_{t}=o⊙\tanh\left(c_{t}\right) \end{array}$$

where σ and *tanh* are the sigmoid function and tanh function, respectively. *W* is the weight matrix. *c*_*t*_, *h*_*t*_, and *x*_*t*_ are the cell state, hidden state, and temporal input at time step *t*, respectively. ⊙ represents element-wise multiplication.

After running the forward of the LSTM network, T hidden state outputs, {*h*_1_, *h*_2_, ..., *h*_*T*_}, are returned and evenly sampled with a 20% probability to enhance generalization capability and avoid overfitting, that is, we uniformly sample T_1_ hidden states from the T hidden states and T_1_ = 1/5 T. Next, the combined hidden states are flattened to the temporal latent features thanks to the subsequent Affine layer to concatenate with the attribute stream. The temporal latent features have a final projected size T_0_ = 5, which is equivalent to putting the temporal latent features into 5 additional latent attribute features. Let's define $h^{t}=H_{1}\left(X^{t}\right)$ as the final 5 latent features of the temporal stream and *x*^*a*^ as the sample values for the original attribute stream *X*^*a*^. The concatenation of these two streams is defined as follows:


(6) $$\begin{array}{l} h^{c}=\Phi \left(h^{t},x^{a}\right)=h^{t}⊕x^{a} \end{array}$$

where ⊕ is the concatenation operator. Then, the concatenated stream *h*^*c*^ is fed into a deep neuron network *H*_2_ which consists of five fully connected layers with number of neurons 1,024, 1,024, 2,048, 1,024, and 1,024, respectively. The output of the model is the predicted probability of being in each disease stage for each sample. Finally, the predicted classification of disease stages *y* is obtained by the following:


(7) $$\begin{array}{l} y=arg maxf\left(\theta ,H_{2}\left(h^{c}\right)\right) \end{array}$$

The network uses *Leaky ReLU* activation function and dropout rate of 30% to enhance the robustness of the model and reduce the computational cost. The learning rate is set to 0.001 and the batch size is set to be the same as the sample size. We use the Adam optimizer, gradient descent algorithm, and softmax cross-entropy loss function to optimize the network.

#### 3.2.3. Uncertainty Quantification of the Model

We perform resampling from our existing samples to quantify the built predictive model's uncertainty. This method is also known as Bootstrap, published by Bradley Efron in Efron (1979). We employ the Bootstrap method because 1) it is invariant under re-parametrization; 2) it does not require the population distribution assumption; 3) it is driven by repeated resampling of data and does not depend on theoretical calculation; 4) it can provide the point estimation and assess the accuracy of the estimation when the traditional statistical method fails.

**Figure 3:**
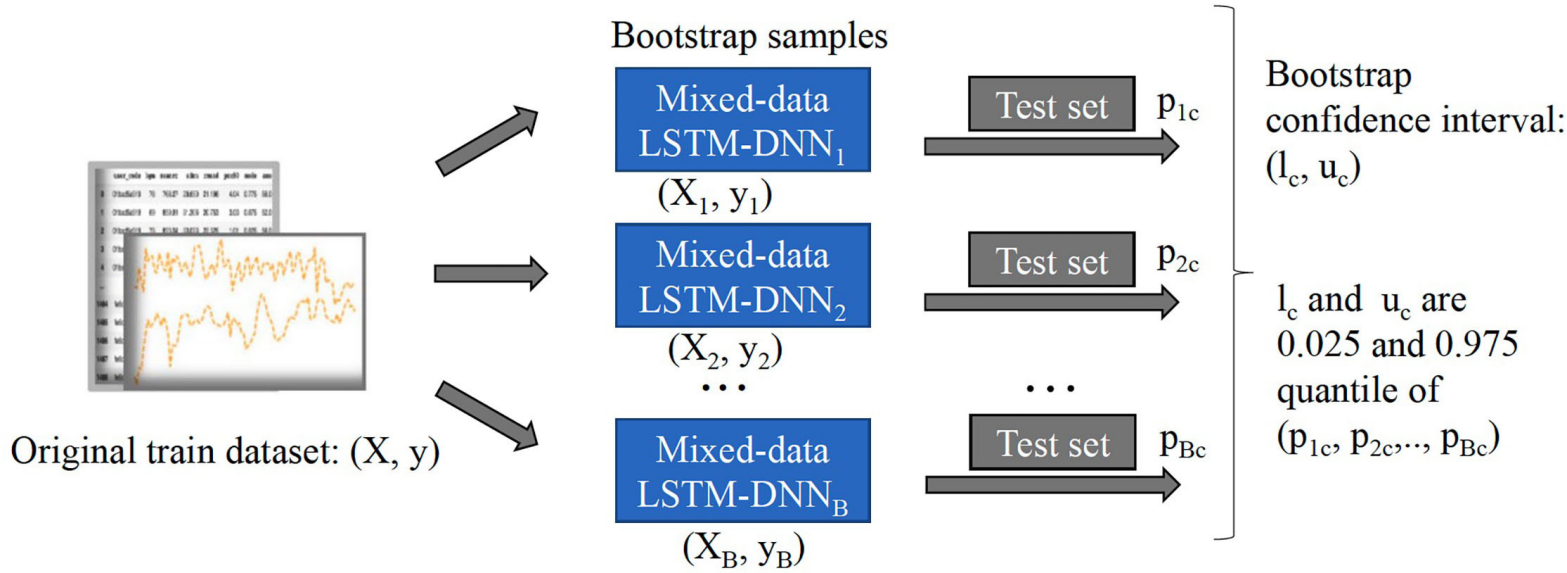
Workflow of bootstrap method to construct 95% confidence intervals.

We present details of the uncertainty quantification algorithm in Algorithm 1. Overall, the intuition of the algorithm is to create new samples, then obtain the prediction output. This process is repeated many times to result in a distribution of output which helps to quantify the model's uncertainty. In order to generate new samples, bootstrapping technique which was introduced by Efron (1979) is utilized. Here, we summarize its workflow in Figure 3:

- Treat the original sample as if it were the population.
- Draw from the sample, at random with replacement, for *B* times (*B* is the number of bootstraps).

Given the value of confidence interval (C.I) α%, we will retrain our model from the newly generated samples, perform classification, and obtain a α% confidence interval of the predicted outcomes.

**Algorithm 1:** Bootstrap method to construct 95% C.I. (Confidence Interval)

function compute_boot_CI ()
**Input:** Input Train dataset *X*, label *y*, Test dataset X^*^, model *f*
**Output:** 95% C.I. (*l*_*c*_, *u*_*c*_), *c* = 1, 2, 3, 4 and *c* is the class index.
For Bootstrap *j* = 1, ..., *B* Generate bootstrap sample *X*_*j*_, *y*_*j*_ from dataset *X* and label *y* with replacement. Train model *f* with bootstrap sample *X*_*j*_, *y*_*j*_. Feed test dataset X^*^ to the above trained model and calculate the prediction outputs *p*_*jc*_, *c* = 1, 2, 3, 4. Let *l*_*c*_ and *u*_*c*_ be the 0.025 and 0.975 percentile of (*p*_1*c*_, ..., *p*_*Bc*_)
**return** (*l*_*c*_, *u*_*c*_), *c* = 1, 2, 3, 4

#### 3.2.4. Baseline Models and Comparison Metrics

To verify the effectiveness and advantages of our proposed approach, we compare the classification results on the test dataset with several classical ML and deep learning models using a five-fold cross-validation approach. The baseline models are as follows:

1. Logistic regression (Logit): a multinomial logistic regression model was used to predict the probabilities of different outcomes for our multi-class problem (Kwak and Clayton-Matthews, 2002).
2. Support vector machine (SVM) (Chang and Lin, 2011): various types of kernels were tried and the kernel with the best result was finally chosen.
3. Attribute-based K-nearest neighbors (KNN) (Peterson, 2009): various number of the *k* nearest neighbors were tried and the *k* with the best result was finally chosen.
4. Long short-term memory: a popular extension of artificial recurrent neural network (RNN) architecture. It was first introduced by Hochreiter and Schmidhuber (1997).
5. Deep Neural Network: it consists of five fully connected layers with a number of neurons 1,024, 1,024, 2,048, 1,024, and 1,024 respectively, and with the same activation function, dropout rate, learning rate, batch size, optimizer, algorithm, and loss function as our model.

For comparison metrics, we use standard metrics such as *accuracy, precision, recall, f1-score*, and *multi-class AUC* (area under ROC curve) to compare the performance of the models. It is worth noting that the inputs to these traditional models above can only be one of the data types and they cannot directly utilize both temporal data and attribute data jointly, so our model is expected to perform better than these models.

### 3.3. Feature Importance

To measure the importance of features, we perform the permutation feature importance algorithm on all the temporal and attribute features in turn to break the relationship between the feature and the true outcome. The permutation feature importance algorithm is described in Algorithm 2. This algorithm is based on our proposed classification model *f*. The general idea is that if a feature is essential for a stage, then shuffling or removing its values increases the model error for that stage because in this case, the model relied on the feature for the prediction. On the other hand, a feature is unimportant for a stage if shuffling or removing its values leaves the model error for that stage unchanged because, in this case, the model ignored the feature for the prediction (Fisher et al., 2019). Therefore, we can rank the losses of the built models after removing one variable at a time to select the most influential features. This approach is applied in Section 4.2 to uncover factors associated with different COVID-19 disease stages.

**Algorithm 2:** Permutation feature importance

function compute_feature_importance ()
**Input:** Feature *X*, label *y*, model *f*
**Output:** Output Feature importance *FI*
Estimate the original model error *e*^*orig*^ = *L*(*y, f*) For feature *j* = 1, ..., *p* Generate feature matrix *X*^*perm*^ by removing feature *j* in the data *X*. This breaks the association between feature *j* and true outcome *y*. Estimate error *e*^*perm*^ = *L*(*y, f*(*X*^*perm*^)) based on the predictions of the permuted data. Calculate permutation feature importance $FI_{j}=e^{perm}/e^{orig}$. Sort features by descending *FI*.
**return** *FI*

### 3.4. Model in a Case Study: Shape-Based Pattern Extraction Model for Signs of Recovery

In the classification of time series, a subsequence is called Shapelets (Ye and Keogh, 2009) if it maximally represents a class in some sense. Grabocka et al. (2014) introduced an implementable method to learn time-series shapelets. In one of our case studies 4.4, we try to find shapelets from HRV data that can differentiate between unrecovered patients and recovered patients. For signs of recovery, the patterns are two groups of shapelets that can linearly separate the recovered from unrecovered patients. Suppose *x*_*i*_, *i* = 1, 2, …, *N* is the *i* − *th* original time series data of length *T*, and *s*_*k*_, *k* = 1, 2, …, *K* is one of the proposed shapelets with length *l*. It is easy to know that in a time series, there are exactly *T* − *l* + 1 segments as long as the starting index of the sliding window is incremented by one. The distance between *x*_*i*_ and *s*_*k*_ is defined as follows:


(8) $$\begin{array}{l} d\left(x_{i},s_{k}\right)=\underset{t\in \left\{1,2,\ldots ,T-l+1\right\}}{\min}{\left\|x_{i,t:t+l}-s_{k}\right\|}_{2}^{2}, \end{array}$$

where *x*_*i, t*:*t*+*l*_ is the subsequence of *x*_*i*_ from time *t* to time *t* + *l*. Since, in our study, the classification task is binary (recovery and unrecovered). Let us define the target variable, i.e., the patient's recovery status *Y*_*i*_, *i* = 1, 2, …, *N*:


(9) $$\begin{array}{l} Y_{i}=\{\begin{array}{cc} 1 & \text{if the }i-th\text{ patient has recovered}\\ 0 & \text{if the }i-th\text{ patient has not recovered}, \end{array} \end{array}$$

Then, the predicted status of the *i* − *th* patient is as follows:


(10) $$\begin{array}{l} \hat{Y_{i}}=W_{0}+\overset{K}{\underset{k=1}{\sum }}d\left(x_{i},s_{k}\right)W_{k}, \end{array}$$

where *W*_*k*_, *k* = 0, 1, …, *K*, are the weights of learning, representing the classification hyperplane. By minimizing the logistic loss function with weight regularization terms, we can learn both the optimal shapelet and the optimal linear hyperplane. The loss function is shown in Equation (11):


(11) $$\begin{array}{l} L=\overset{N}{\underset{i=1}{\sum }}l\left(Y_{i},\hat{Y_{i}}\right)+\lambda {\left\|W\right\|}_{2}^{2}, \end{array}$$

where


(12) $$\begin{array}{l} l\left(Y_{i},\hat{Y_{i}}\right)=-Y_{i}log\sigma \left(\hat{Y_{i}}\right)-\left(1-Y_{i}\right)log\left(1-\sigma \left(\hat{Y_{i}}\right)\right), \end{array}$$

and σ is the sigmoid function.

In the optimization process, a stochastic gradient descent (SGD) approach is adopted. Note that because SGD needs all the functions to be differentiable, an approximation of the minimum function (8) is used. This function is called the Soft Minimum function (Grabocka et al., 2014) and is shown in Equation (13).


(13) $$\begin{array}{l} \hat{d}\left(x_{i},s_{k}\right)=\frac{\sum_{t=1}^{T-l+1}d_{i,k,t}e^{\alpha d_{i,k,t}}}{\sum_{t^{\prime }=1}^{T-l+1}e^{\alpha d_{i,k,t^{\prime }}}}, \end{array}$$

where


(14) $$\begin{array}{l} d_{i,k,t}={\left(x_{i,t:t+l}-s_{k}\right)}^{2}. \end{array}$$

By applying the above method to the patient's HRV time series data, we aim to find a sequence pattern that can show signs of patient recovery to the greatest extent possible. Our results are shown in Section 4.4.

## 4. Experimental Results

### 4.1. Infected Stage Classification Performance Evaluation

**Figure 4:**
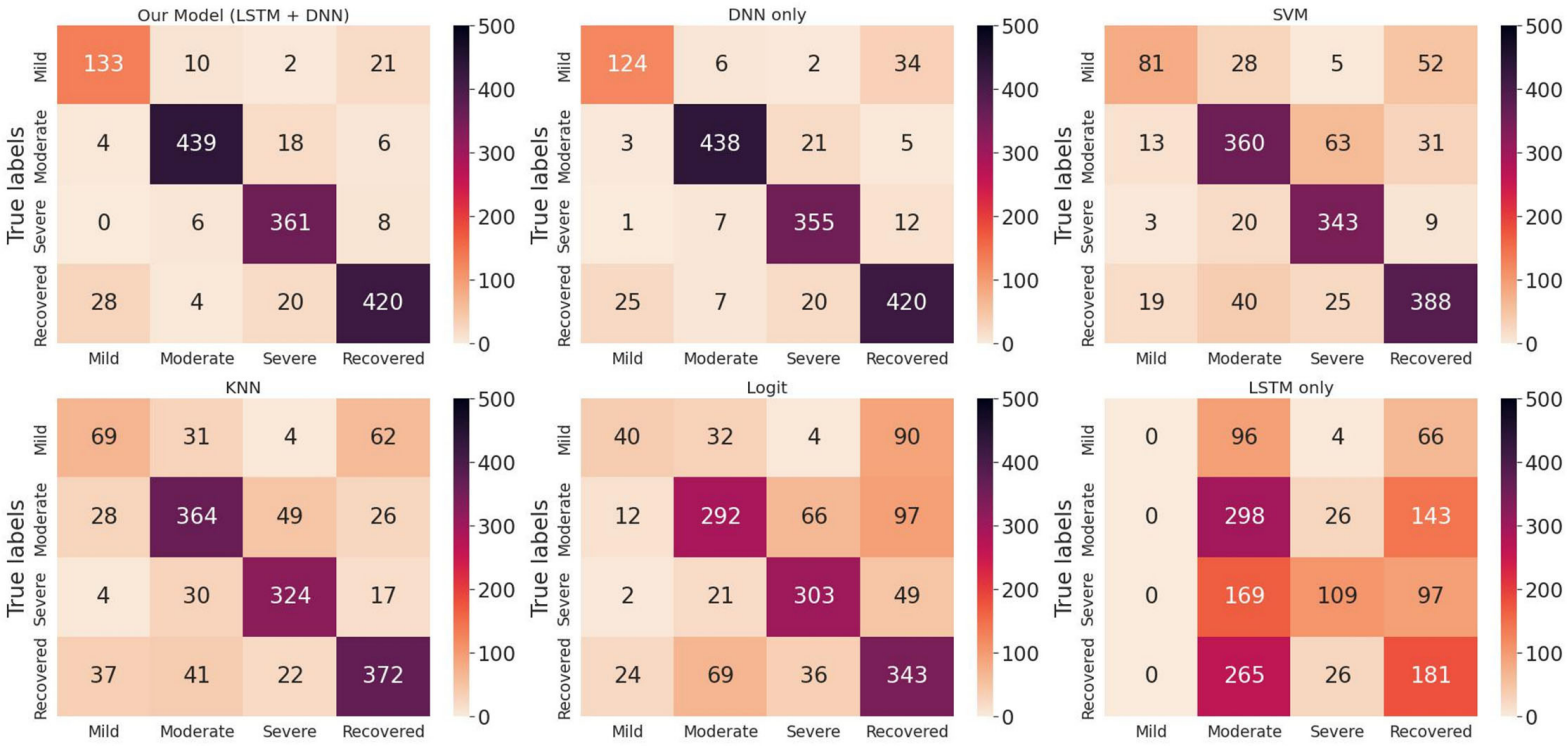
Total confusion matrix for COVID-19 disease stage classification based on 5-fold cross-validation.

We randomly split up the data prior to modeling so that all models can use the same data splits. Each time, the models are trained on 4-folds (80% of the data) and tested on 1-fold (20% of the data). These 5-folds take turns being the test dataset to ensure that each sample can be classified. We perform a comprehensive comparison of model classification results. We add up the confusion matrices of the five experiments to obtain the total confusion matrix, which is therefore based on the result of all samples, as shown in Figure 4. For the five evaluation metrics, accuracy, precision, recall, f1-score, and multi-class AUC, we use the average results of the five experiments as the final evaluation results, which are listed in Table 3.

**Table 3:** Infected stage classification results of models based on 5-fold cross-validation.

**Model**	**Accuracy**	**Precision**	**Recall**	**F1-score**	**AUC**
LSTM Only	0.397	0.410	0.397	0.355	0.556
Logit	0.661	0.666	0.661	0.650	0.741
KNN	0.763	0.761	0.763	0.759	0.816
SVM	0.792	0.791	0.792	0.787	0.839
DNN Only	0.903	0.905	0.903	0.903	0.924
Our Model (LSTM+DNN)	**0.914**	**0.917**	**0.914**	**0.914**	**0.935**

*The bold values indicate the best result for each metric*.

On the one hand, we can see the improvement in classifications of our proposed model from the confusion matrix. Our model has less misclassification of disease stages compared to other models. On the other hand, the detailed results in Table 3 also show the advantages of our model. To be specific, the three models Logit, KNN, and SVM are comparable, having accuracy scores of about 0.66 to 0.79 and AUC of about 0.74 to 0.84. The LSTM model gives poor results due to the fact that it only uses temporal data. DNN model is the second-best model with an accuracy score of 0.903 and AUC of 0.924. Our proposed method has the highest scores under all five metrics, with an accuracy score of 0.914 and AUC of 0.935.

**Figure 5:**
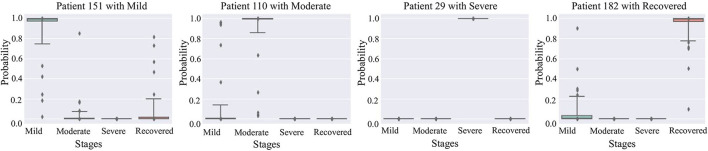
95% confidence interval of the prediction probabilities for the current stage of COVID-19 patients.

Figure 5 are box plots that present uncertainty quantification of the disease stage predictions of our proposed model for some randomly selected patients (Patient 151, 110, 29, and 182). The narrow box plot indicates the narrow 95% C.I., which presents low uncertainty in the prediction. We observe that for patient 29, all the C.I.s are quite narrow, while for all other patients, the C.I.s for certain stages are wider, which shows high prediction uncertainty. Even though there is high uncertainty in the prediction of certain disease stages, the 95% CI for each stage classification has shown that the probability of the classified stage (final prediction on each patient) always has a higher probability value than other stages. It means that our predictive model successfully identifies the disease stages with the performance results provided in Table 1.

### 4.2. Uncovering Factors Associated With COVID-19 Disease Stages

**Figure 6:**
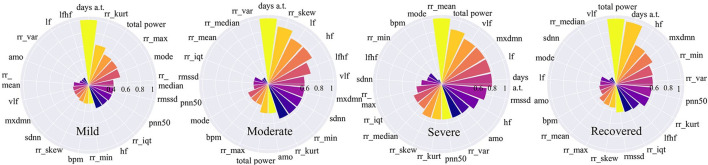
Feature importance for COVID-19 stages. The higher the value is, the more important the feature is.

In this section, we focus our analysis on features from wearable data instead of other factors which have been discussed through news channels such as background diseases or body symptoms. We use a random permutation of values shown in Algorithm 2 to calculate feature importance values for each feature based on the ratio of the model's errors between permutations. After obtaining the importance values, these values are rescaled to the range [0–1] to make them comparable. The results are shown in Figure 6. For each stage, the important features are ranked from high to low. The high importance feature means that prediction performance is highly dependent on this feature.

Figure 6 shows that for mild and moderate stages, the number of days from onset symptoms (*days a.t*.) is the most important since it ranks top among all variables. It means for mild and moderate patients, HRV variables have not yet shown very obvious characteristics, while the number of sick days can best determine the patients at this stage. This phenomenon is more reliable for mild patients since the number of sick days is far more important than the second-ranked variable. This result can be explained that in the early days of COVID-19 infection, most people have mild symptoms. For severe patients, the number of sick days is no longer important, the average time between each heartbeat, *rr*_*mean*, occupies the most important position, even though it is very unimportant in other stages. It indicates that the *rr*_*mean* of severe patients is very different from those patients in other stages. In other words, if the condition of a patient gets worse, it will be most clearly reflected by *rr*_*mean*. For recovery patients, the total power of waves generated by the heart (*total*_*power*) and the number of sick days (*days a.t*.) are important variables. This shows that, on the one hand, it takes a certain number of days for patients to recover; on the other hand, a significant change in the total power of the waves generated by the heart is most indicative of the recovery phase.

If we focus on different frequency wave power generated by the heart (high-frequency: *hf*, low-frequency: *lf*, very-low-frequency: *vlf*), we can also find something valuable. In the mild stage, no such variables are important. However, in moderate stage, the importance of all the three along with the ratio of low to high frequency waves (*lfhf*) rank relatively high. Therefore, compared to the patients in the mild stage, the wave power of each frequency of patients in the moderate stage has changed obviously. Besides, for severe patients, the frequency waves that are most different from other stages are low-frequency waves (*vlf*, *lf*). While for recovered patients, the frequency wave that is most different from other stages is a high-frequency wave (*hf*).

### 4.3. Case Study: Severe Patients vs. Mild Patients

**Figure 7:**
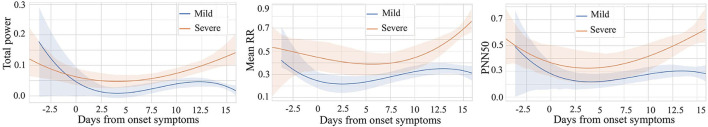
Comparison of mild vs. severe patients based on three variables: *Total power, Mean RR*, and *PNN50*.

Since heart rate variability (HRV) is popular in many healthcare-related research, we chose to explore it to compare daily patterns of severe patients vs. mild patients. The variables for comparison are the average time between each heartbeat (*rr*_*mean*), the percent of RR-intervals that fall outside a 50 ms range of the average (*pnn*50), and the total power of high-frequency waves, low-frequency waves, and very-low-frequency waves generated by the heart (*total*_*power*). All the data is normalized with the min-max technique to make them comparable. In addition, we choose data from 5 days before the onset of symptoms to 16 days after the onset of symptoms to show the difference between different stages in the most critical time. We use polynomial regression to do curve fitting and trending analysis separately. At the same time, 95% confidence intervals of fitted curves are shaded. We can find something interesting in the results shown in Figure 7.

We noticed that the highest value of the *total*_*power* curve and its confidence interval did not exceed 0.3. This range of *total*_*power* is relatively narrow since we have scaled all the data to the unit interval. It indicates that for people who have COVID-19 symptoms, whether he or she is in the mild stage or the severe stage, the total power of waves generated by the heart is lower approximately a few days before and 2 weeks after the onset. For these three comparative variables, *rr*_*mean*, *pnn*50, and *total*_*power*, their curves have a similar pattern. In general, after the symptom onset date, all three variables of severe patients are higher than those of mild patients. The higher value of average time between each heartbeat of severe patients means that their average heart rate is slower than that of mild patients. Furthermore, severe patients usually have higher *pnn*50. In other words, for severe patients, the outlier heartbeats, heartbeats whose intervals are farther apart from the average interval, occupy a larger proportion. It reveals that the heart rhythm of severe patients is more irregular than that of mild patients. Besides, compared to mild patients, heart-generated wave power of severe patients is stronger.

Following the time dimension, we can also find the different development of the above variables during the illness of mild and severe patients. Curves of patients in severe stage show a trend of increasing after decreasing. The curve of patients in mild stage also decreases at the beginning, while gradually stabilized after the curve rose and then again has a decreasing trend at about 12 to 14 days. This may be because the immune regulation of mild patients does not allow them to rise endlessly, which may also be a feature of gradual recovery. We can also see that after about 13 days, the 95% CI of the curves of both severe and mild patients are relatively narrow, which gives us more confidence to believe that severe and mild patients have indeed evolved in two directions.

### 4.4. Case Study: Signs of Recovery

In this case study, we try to find the most discriminate patterns that classify best the recovered stage and other stages. These patterns will signify the sign of recovery instead of the progressing disease. In addition, HRV data for the evening hours is used for analysis to avoid the influences of daytime activities of patients. We use the HRV sequence variables, which are the interval between consecutive heartbeats(*RR*), its lag difference sequence(*DI*), and its sequence of absolute deviation from the mean(*DM*) to extract the patterns. The methods for creating *DM* and *DI* can be found in Section 3.1.2. All three time series are normalized and combined to explore the discriminate patterns of recovery signs (See Section 3.4).

**Figure 8:**
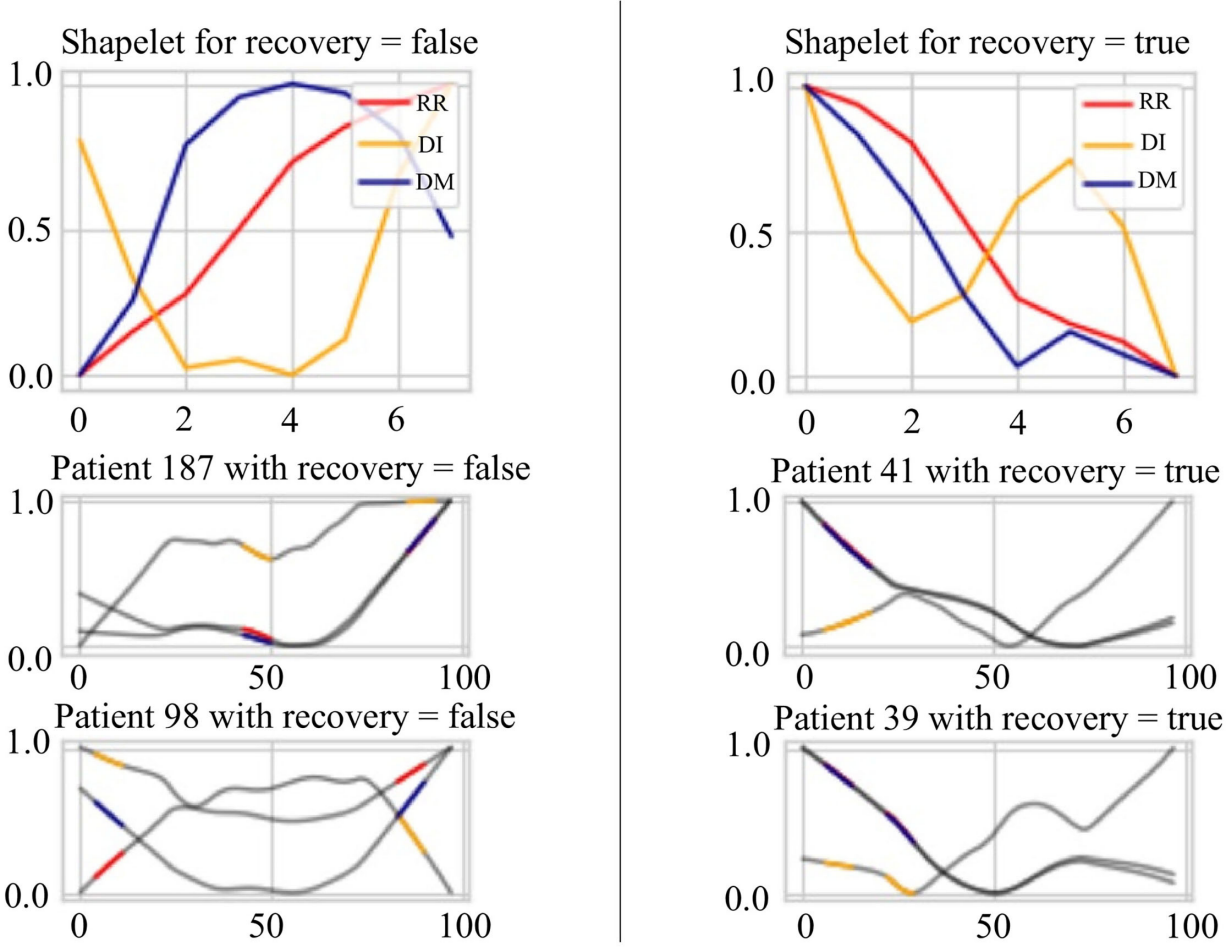
Signs of recovery. The left and right columns present time series shapelets that differentiate between unrecovered patients and recovered patients, respectively. The red shapelet (RR) is the original heartbeat interval. The yellow shapelet (DI) is the differencing transformation of the heartbeat interval. The blue shapelet (DM) is the deviation of the heartbeat interval from the mean value.

Figure 8 presents the extracted patterns that best discriminate the sign of recovery (top two subplots) and sample patterns from the patients (bottom four subplots). First, the heartbeat interval data *RR* (in red) shows a decreasing trend for recovery cases than an increasing trend for other stages. Second, the heartbeat interval differencing data *DI* (in yellow) shows a sine-shaped pattern in the recovered group while it is a concave-parabola shape for unrecovered samples. Last, the absolute deviation from the mean data *DM* (in blue) shows a gradually decreasing trend in the recovered stage compared to a convex parabola shape in unrecovered situations. We can conclude a frequent change from these shapelets and an inconsistency of the COVID-19 patients. On the other hand, it shows an overall decreasing trend of the HRV data for the recovered patients in the evening. The subplots of the four patients show portions highlighted by different colors representing different time series. These portions are the ones that are closely similar (having short Dynamic Time Warping (DTW) (Sakoe and Chiba, 1978) distance in latent space) to the extracted shapelets and contribute to identifying signs of recovery.

## 5. Limitation

There are a few limitations in our study coming from the selected dataset. The number of patients in the study is 186, and they are not randomly selected. So, they are not representative of the entire population. However, this situation usually happens in healthcare data science research since it is time-consuming and expensive to obtain full data from a large population for the initial study. In addition, the uncertainty quantification of the model is down with the assumption that the set of observations is from an independent and identically distributed population. Moreover, some of the recorded data like coughing, having diabetic disease, etc., are self-reported, which have their own limitation. Self-reported information may not be accurate, depending on how honest the patients were when they did the survey.

## 6. Conclusion

In this work, we propose a novel predictive model to categorize COVID-19 patients into multiple stages (mild, moderate, severe, and recovered), using a wearable device dataset. Our predictive model exploits temporal stream data and attribute stream data simultaneously for disease stage classification and is able to identify severe patients in an earlier stage even if the symptoms seem to be “mild” or “moderate.” In addition, we apply bootstrap methods to perform uncertainty quantification for the predictive model, and the experimental results demonstrate our predictive model's higher classification accuracy than other existing baseline approaches. Furthermore, we investigate each feature's importance to uncover its association with COVID-19 using a model-agnostic approach. Lastly, we investigate two cases in detail: 1) the first one is used to illustrate the comparisons between mild patients and severe patients. 2) the second one is used to analyze the signs of recovery. We observe that there are fluctuating HRV patterns in severe patients, but a more stable pattern and a clear trend in mild patients or recovering patients.

## Data Availability Statement

The original contributions presented in the study are included in the article/supplementary material, further inquiries can be directed to the corresponding author.

## Ethics Statement

Ethical review and approval was not required for the study on human participants in accordance with the local legislation and institutional requirements. The patients/participants provided their written informed consent to participate in this study.

## Author Contributions

MG contributed to the model design and performed experiments. LN contributed to the experimental design and wrote the first draft of the manuscript. HD contributed to the model performance evaluation. FJ was responsible for the overall supervision and experiment design. All authors contributed to manuscript revision and provided critical feedback and helped shape the research.

## Conflict of Interest

The authors declare that the research was conducted in the absence of any commercial or financial relationships that could be construed as a potential conflict of interest.

## Publisher's Note

All claims expressed in this article are solely those of the authors and do not necessarily represent those of their affiliated organizations, or those of the publisher, the editors and the reviewers. Any product that may be evaluated in this article, or claim that may be made by its manufacturer, is not guaranteed or endorsed by the publisher.

## Footnotes

^1^ https://en.wikipedia.org/wiki/Coronavirus_disease_2019

^2^ https://www.fitbit.com

^3^ https://github.com/Welltory/hrv-covid19
